# User evaluation of a novel SMS-based reminder system for supporting post-stroke rehabilitation

**DOI:** 10.1186/s12911-019-0847-3

**Published:** 2019-07-03

**Authors:** Uno Fors, Julius T. Kamwesiga, Gunilla M. Eriksson, Lena von Koch, Susanne Guidetti

**Affiliations:** 1 Department of Computer and Systems Sciences (DSV), Stockholm University, Stockholm, Sweden; 2 Uganda Allied Health Examinations Board, Kampala, Uganda; 3 Division of Occupational Therapy, Department of Neurobiology Care Sciences and Society, Karolinska Institutet, Stockholm, Sweden; 4 Department of Neuroscience, Rehabilitation Medicine, Uppsala University, Uppsala, Sweden; 5 Theme Neuro, Karolinska University Hospital, Stockholm, Sweden

**Keywords:** Stroke, Rehabilitation, Client-centred, Feedback, Occupational therapy, SMS-reminders, Africa

## Abstract

**Background:**

According to WHO stroke is a growing societal challenge and the third leading cause of global disease-burden estimated using disability-adjusted life years. Rehabilitation after stroke is an area of mutual interest for health care in many countries. Within the health care sector there is a growing emphasis on ICT services to provide clients with easier access to information, self-evaluation, and self-management. ICT-supported care programs possible to use in clients’ home environments are also recommended when there are long distances to the health care specialists.

The aim of this study was to evaluate the technical usability of a SMS-based reminder system as well as user opinions when using such a system to assist clients to remember to perform daily rehabilitation activities, to rate their performance and to allow Occupational therapists (OT’s) to track and follow-up clients’ results over time.

**Methods:**

Fifteen persons with stroke were invited to participate in the study and volunteered to receive daily SMS-based reminders regarding three activities to perform on a daily basis as well as answer daily SMS-based questions about their success rate during eight weeks. Clients, a number of family members, as well as OTs were interviewed to evaluate their opinions of using the reminder system.

**Results:**

All clients were positive to the reminder system and felt that it helped them to regain their abilities. Their OTs agreed that the reminder and follow-up system was of benefit in the rehabilitation process. However, some technical and other issues were limiting the use of the system for some clients. The issues were mostly linked to the fact that the SMS system was based on a Swedish phone number, so that all messages needed to be sent internationally.

**Conclusion:**

In conclusion, it seems that this type of SMS-based reminder systems could be of good use in the rehabilitation process after stroke, even in low income counties where few clients have access to Internet or smart phones, and where access to healthcare services is limited. However, since the results are based on clients’, OTs’ and family members’ expressed beliefs, we suggest that future research objectively investigate the intervention’s beneficial effects on the clients’ physical and cognitive health.

**Electronic supplementary material:**

The online version of this article (10.1186/s12911-019-0847-3) contains supplementary material, which is available to authorized users.

## Background

### About stroke

According to the World Health Organization (WHO) stroke is a growing societal challenge and is the third leading cause of global disease-burden estimated using disability-adjusted life years [1]. Stroke causes impairments, activity limitations and participation restrictions [2] which often result in decreased functioning in everyday life. The increasing number of people having stroke leads to a growing global demand for rehabilitation services but is especially true in low- and middle-income countries in which a significant number of people have stroke [3]. However, the availability of rehabilitation services is scarce in many regions of the world. Rehabilitation after stroke is therefore an area of increasing importance for healthcare in many countries, including Sweden and Uganda.

The increasing burden of stroke but limited access to rehabilitation services creates a need for developing new strategies such as the use of Information and Communication Technologies (ICT) like mobile phones for provision of healthcare services [4]. Within the healthcare sector there is a growing emphasis on ICT-based services to provide clients with easier access to information, self-evaluation, and self-management. ICT-supported programs in clients’ home environment are also recommended when there are long distances to the health care specialists [5].

### About rehabilitation of stroke and the need for reminders

The goal for rehabilitation of people with stroke is defined as increased functioning and participation in life, (i.e. body function, activity and participation) and well-being [6]. One way of reaching this goal is to focus on increasing the ability and independence in activities in daily living (ADL). Evidence is weak for various general rehabilitation interventions as interventions for improved motor functioning. However, there is strong evidence for task-specific training, meaning activities or tasks that are relevant and purposeful for the individual [7], as well as for ADL interventions [2, 8]. Therefore, activities that are perceived as relevant and purposeful in everyday life for persons with stroke can be used as goals in an intervention to improve the ADL functioning [8].

Interventions for compensation for the impact of cognitive disabilities, with the goal to improve the performance of ADL is common within stroke rehabilitation [9, 10] and assistive devices with for example reminders have contributed to improving the performance of activities among people with cognitive disabilities as memory problems in the subacute phase as well as in the long term [9, 11].

### Reminder systems for clients

Computer based reminder systems have been tested and found to be of good value in other domains than stroke. For example, Dexter et al. [12] described a reminder system to increase the use of preventive care for hospitalized clients, which lead to a significant increase of such measures. Jangi et al. [13] performed a systematic review on reminder systems used in physical therapy, which showed that reminders in the form of SMSes, phone calls, letters or e-mails could have a good effect on improving clients’ adherence to physical therapy exercise programs. However, there have also been studies that indicate that automated reminders might not be superior to paper based methods for clients with stroke [14].

### ICT-based interventions for stroke

A number of different ICT-based systems have been proposed and tested to assist with the rehabilitation after stroke. For example, computer-support in terms of Telehealth solutions [15], Robotics [16], Virtual Reality [17, 18] as well as Off-the-shelf computer games [19] have been tested. Also, more complex models with home-based ICT-platforms have been applied [20].

However, most of these ICT-based solutions have been focused on a more direct training and rehabilitation of motor functions. Few have targeted the basic idea of reminding the clients, and in a positive way, challenging the clients to perform their recommended daily activities using ICT-tools. Even fewer of such studies have investigated the use of such systems in developing, less wealthy, regions of the world.

Smart-phones have been used within rehabilitation for example in India where modules with information about stroke, exercises to be performed at home, training of functional skills as well as of ADL, and the use of assistive devices have been provided to persons with stroke and their family members. This information was provided through text and video-clips on smart phones and were field-tested and found feasible and acceptable by the persons with stroke and their family members [21]. Interventions with the use of short message services (SMS) has been found effective to improve medication adherence after stroke [22], but studies which use SMSes as reminders for performing activities as part of a rehabilitation program after stroke have not been found.

### Stroke and rehabilitation in Uganda

In Uganda, despite the large number of stroke cases, there are very few occupational therapists (OTs) and rehabilitation resources available. Therefore, most clients have so far been left to the care of their families and with very limited professional assistance [23].

Moreover, even if the use of the internet and mobile phones have dramatically increased also in many low income countries, a majority of the users in Uganda still use more basic, non-smart phones with very limited or no Internet connection, leading to special challenges in using internet-based high tech solutions in healthcare services.

### About the overall F@ce project

This project is a sub-study of the overall stroke rehabilitation project *“Participation in daily activities in everyday life after stroke - Developing and evaluating a model for a mobile phone supported and client-centered rehabilitation intervention in Uganda”* led by researchers at Karolinska Institutet, Sweden*.*

The Medical Research Council’s guidance for developing complex interventions [24], has been used in previous studies of a client-centred, activities in daily living (CADL) intervention, according to Bertilsson et al. [25]. Based on the theoretical base of an occupational and phenomenological perspective as well as the rationale of CADL, with its different components, a further development involving a qualitative study of how persons who have had a stroke used their mobile phones [26] as well as a culturally adapted Stroke Impact Scale (SIS) 3.0 has previously been performed in Uganda [27]. This rehabilitation intervention (called F@ce™) has been further developed and refined in collaboration with experienced practitioners, researchers and health informaticians.

F@ce stands for Face-to-face (F) with a collaboration between the therapist and a client during all the different phases, including Assessment (@), Collaboration (C) and Evaluation (E). The F@ce™ intervention integrates the principles of client-centred practice with goal setting involving daily occupations that the person need and want to do in everyday life during the entire rehabilitation process and has in a feasibility study been found to improve primary outcomes (performance and satisfaction of valued daily activities in everyday life assessed using the Canadian Occupational Performance Measure (COPM), as well as self-efficacy [28].

To support the work according to F@ce™, a SMS-based reminder and client monitoring tool was developed. This sub-study focus on the technical usability in terms of features and functions of the sub-systems for managing, sending and receiving SMS-reminders as well as on the user opinions of this health informatics solution.

### Aim

The aim of this study was to evaluate the technical usability (as described above) and user opinions when using a SMS-based reminder system to assist clients to remember to perform daily rehabilitation activities, to rate their performance and to allow OT’s to track and follow-up clients’ results over time.

## Methods

### Study setting

This study was a sub-study of a larger overall clinical study (F@ce), described elsewhere [28]. The study was a collaborative project between Karolinska Institutet (KI) in Stockholm (Division of Occupational Therapy), Sweden; Stockholm University, Sweden (SMS-system development and related services); and Uganda Allied Health Examinations Board (coordination of local OTs).

The overall study focusing on the rehabilitation of post-stroke clients in Uganda was based on a set-up with four phases starting with 1. A training workshop for the OTs that should deliver the intervention according to F@ce™; 2. Gathering and informing subjects and local OTs and collecting base-data from all clients; 3. The F@ce™ intervention, including formulating three individual targets (goals) and planned strategies for recapture the target activities; 4. Collecting final data including interviewing clients and their relatives. This sub-study was focusing on the methodology of using SMS-based reminders to support clients to perform the daily training activities (goals).

All activities in Uganda were coordinated by a local OT (JTK), who was in direct contact with the other three OTs. The whole project was governed by a group of Swedish OT researchers. JTK was a PhD student at KI during the time of the study.

### Participants

Three OTs volunteering to participate in the overall study were trained in a workshop in Kampala, Uganda February 2016 to deliver the intervention according to F@ce™ and use an SMS-based reminder system to support the rehabilitation of their clients.

In the overall clinical study [28], thirty persons were invited post-stroke by the local OT to, on a voluntary basis, participate in the study. The overall study was set-up with a quasi-experimental pre-post design with an intervention group (IG, *n* = 15) receiving the F@ce™ intervention using SMS-based reminders and a control group (CG, n = 15) where no SMS was supplied during the study period.

Since this actual sub-study was focusing on the technical usability and user opinions of the SMS-reminders, only the 15 clients in the IG were included here.

All participants had been diagnosed with stroke and were living in or adjacent to the capital of Uganda, Kampala.

Eleven of the family members of the participants in the intervention group were also asked to participate in an open-ended interview on their experiences of living with a person with stroke and experiences of the F@ce™ intervention and their opinions of using the SMS-system.

### The SMS reminder system

A Web-based system for managing client data, entering their and the OTs phone numbers, the three targeted daily activities per person who have had a stroke, as well as the timing for reminders and other data collection, was developed using Node.js/PostgreSQL as a backend and HTML/CSS/JavaScript as a front-end.

The central SMS-system was in its turn connected to an international commercial SMS API service called Twilio™ (Twilio.com). This was due to the fact that the project management could not find a local tele-operator in Uganda who could give the same service. This entailed that all SMS needed to be sent from and to a Swedish phone number.

**Fig. 1:**
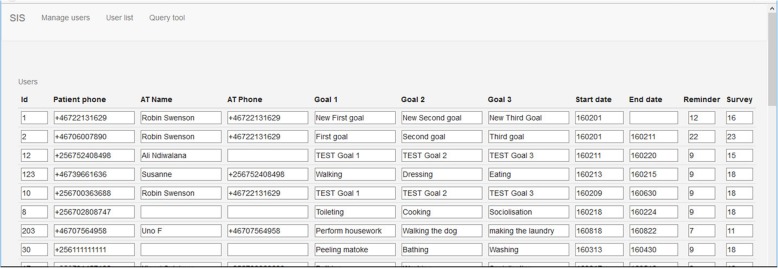
The SMS client management system main interface. Note that these are not real client data

The SMS-system allowed the local OT team to formulate individually targeted daily training activities that were designed to support the persons to improve their activities of everyday life according to the principles of F@ce™. Due to the limitations of the SMS-technology, all reminders needed to be formulated very briefly, like “washing laundry”, “swipe the compound” etc. However, the local OTs could in the discussions with the clients explain all three daily activities in more detail, if needed. The SMS-system was also designed to assist the OTs to manage all client data, including mobile phone numbers and individual daily activity targets and when to send out the morning SMS reminder and the evening follow-up questions. Please refer to Fig. 1 for a screen-shot of the central system.

**Fig. 2:**
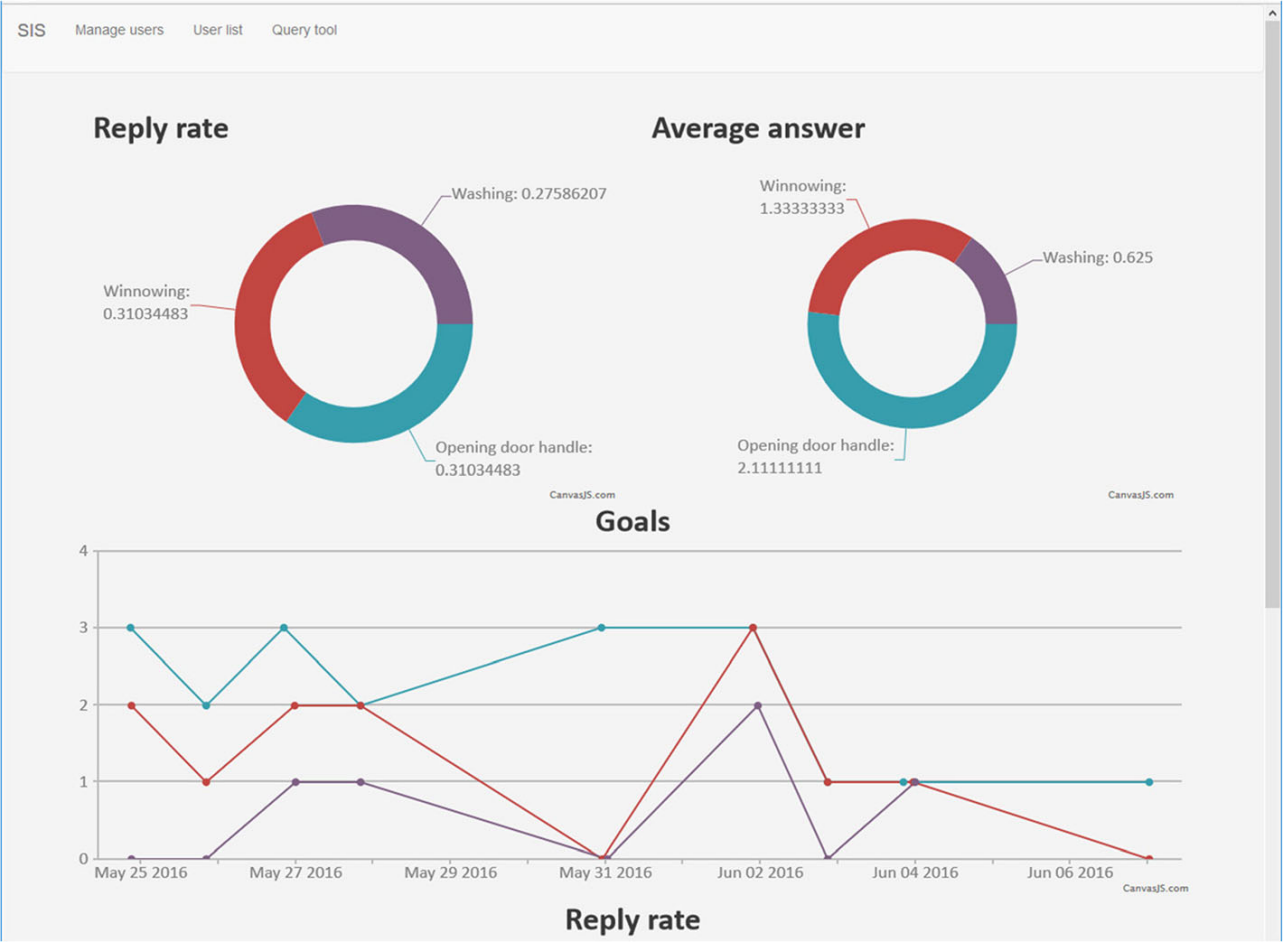
A screenshot of some of the statistics the central SMS client management system may present. Note that these are not real client data

The system could also follow-up clients over time and display statistics of their responses to facilitate the monitoring of the clients’ daily activities and possible success in doing them, see Fig. 2. The system could also display time-series graphs for each individual client or groups of clients. This feature could also be used for gathering follow-up data for the research team.

**Fig. 3:**
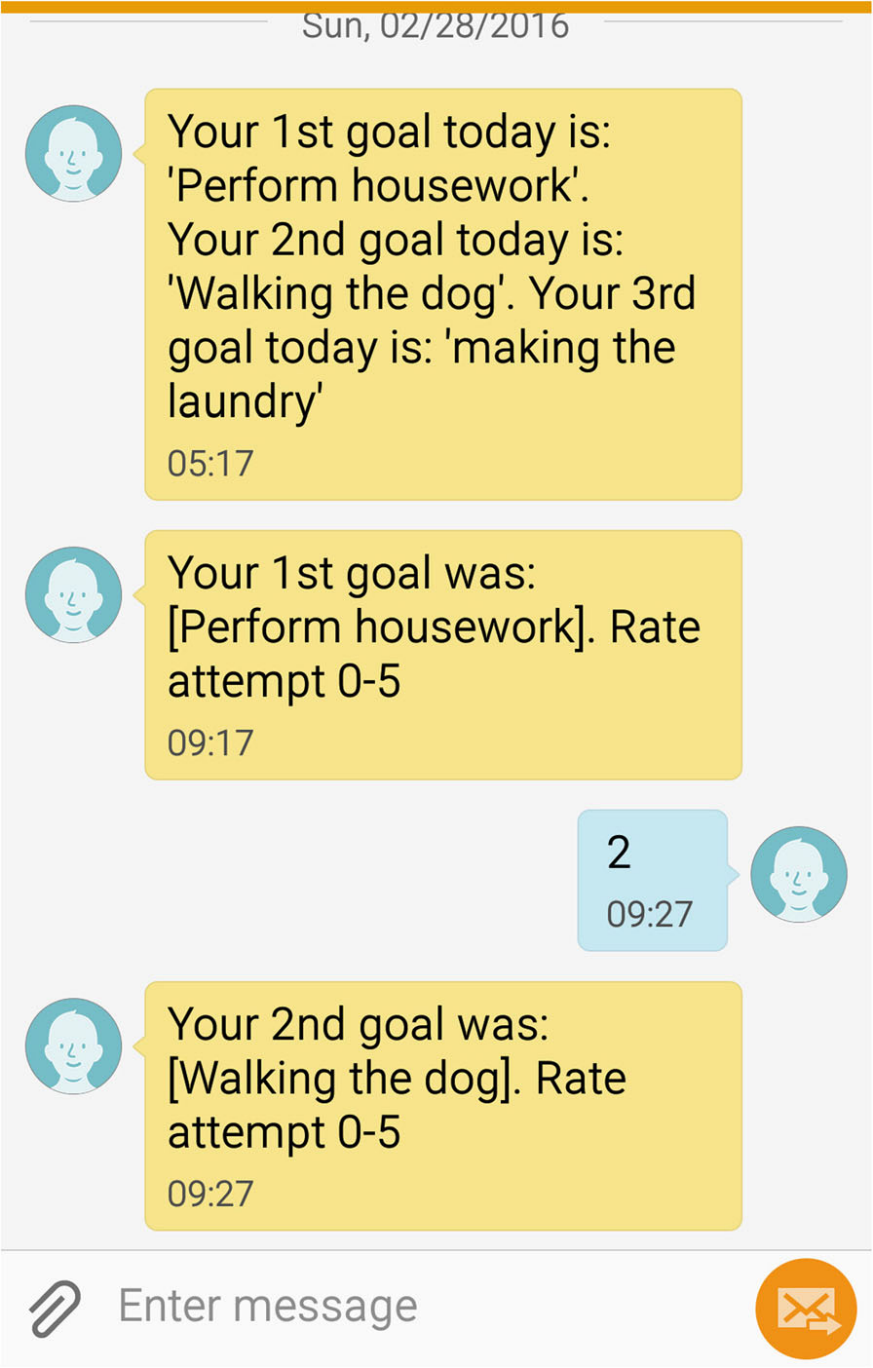
A screenshot from a hypothetical client’s phone showing the morning SMS with the three activities and two of the evening SMS questions of success rate. Note that these are not real client data

The management system was used according to the following procedure:

1. The OT recruits a client, informs about the project and, together with the client, decide on suitable activities to reach the targets.
2. The OT enters client’s phone number, his/her own phone number, the three activities, period of the reminders and time per day for sending out the SMSes with reminders of the activities, and time per day to send out questions regarding the success rate of the activities in the system.
3. Each morning, the individual client receives one SMS reminder of the three target activities, see Fig. 3. If the client could not manage the mobile phone, a family member was to receive the SMS, support the client to rate the performance, send scores by SMS and encourage the client to perform the activities.
4. Each afternoon the client receives three different SMSes with a question per target activity, where the client is supposed to rate the level of success of the activity, see Fig. 3. The clients were instructed to indicate that if he/she did not perform that specific activity they should answer with a 0 (meaning “has not performed the activity”), or rate how successful they were (where 1 mean “not so good”, and 5 “carried out the activity very well”). Participants who rated 0 or who did not reply to the SMS reminder message, automatically launched a red flag on the OT’s mobile phone. The OT would then call the participant the following morning to find out what had happened.
5. Steps 3 and 4 were repeated every weekday during the study period (8 weeks).
6. After the study period, the local OT interviewed the clients about their experiences of using the SMS-system as a part of their rehabilitation.

The messages that were sent to the clients were based on pure text SMS-messages due to the issue that most people in Uganda do not have a Smartphone. In our study, only one participant of the 15 in the SMS-group had a Smartphone.

Since we expected that most of the clients had an financial situation where sending and receiving daily SMSes from and to Sweden was too expensive, the project bought “Air-time” for all participating clients so that sending and receiving SMSes were free of charge during the study period.

During the first meeting, the local OT informed about stroke and provided advice to promote independent functioning in ADL. Some participants, also might have received other rehabilitation services as needed, e.g. physiotherapy and speech therapy.

The central SMS system automatically kept track of each clients’ activities and results. If a client response was a “0” in any of the three activity questions or if he/she did not reply at all, the associated OT automatically received a SMS with instructions to contact that individual client and check what the problems might be.

### Interviews with participants

After the 8 weeks all participating clients were approached by the coordinating local OT (JTK) who also performed the post-intervention assessments of the overall study [28]. In this context and when that data collection was completed, the participants were interviewed about their experiences of the F@ce™ intervention as well as the SMS-services. Both the clients and their family members were interviewed. Altogether 22 participants were interviewed individually: 11 clients (two clients dropped out from the intervention and two other could not be reached at the time for the interviews) and 11 family members. The questions were semi-structured and adhered to an interview guide developed by the authors. A number of the questions in the interview guide focused on the technology of the intervention, for example: During these 8 weeks of intervention; What do you think worked well in the intervention? Can you please tell us what you think about the technology used, the SMS? Have you experienced any problem with the technology? How has it been for you rating the goals using SMS? Each interview lasted roughly between 40 to 60 min. The participants were thus asked about their experiences regarding the intervention, its impact on their daily life and the SMS-service and its advantages and challenges. Their answers were recorded digitally and then transcribed. Some answers were in Luganda and those interviews were first transcribed and then translated into English. The verbatims on the experiences of the intervention and how it impacted their daily life were then analysed using latent content analysis [29]. The clinical part of the interviews will be presented in a future publication. The part of the transcripts revealing the experiences of the use of the mobile phone and the SMS-service were read through and the participants’ descriptions were sorted according to whether they were positive or negative to the SMS services. The content analysis as well as the analysis of the narrated experiences of the SMS-service was performed by the second, third and last author.

### Interviews with the OTs

The local main OT in this project was coordinating the project and was also responsible to instruct and follow-up the work by the three other OTs. All OTs were also interviewed regarding their opinions regarding the technical usability and clinical possibilities of using the reminder system.

Additionally, since the local main OT was in regular contact with the other OTs, he received feedback from the OTs on a regular basis where they reported smaller or larger issues of the SMS reminder system and the rehabilitation process. The OTs also filled in information in their log books.

In addition, the three assisting OTs and the local main OT also answered a questionnaire. The questions asked them to rate advantages and challenges involved in using the SMS-service. See Additional file 1 for details.

## Results

All 15 clients agreed in collaboration with their OTs on three daily target activities. The most common activities were washing clothes, sorting beans, dressing self, but many other activities were also targeted. None of the clients complained about that the very brief SMS-descriptions of the daily activities (e.g. “washing laundry”) were difficult to follow.

Two clients in the intervention group dropped out because they moved out from the Kampala region and could not be reached by the research team. The remaining 13 clients continued to use the SMS throughout the 8 weeks. However, at the time for the interviews, two of the clients could not be reached, ending up in 11 clients interviewed totally.

The SMS system for reminders worked rather well during the project, however due to unknown reasons one client’s phone was unable to receive our SMSes during a period. An unanticipated issue was also that some of the participating clients were affected by their stroke so that they could not operate their own phones, but was relying on a family member to receive the reminders and send the evening rated responses. Furthermore, some clients did not use their phones on their own, but asked a family member to gather the SMSes and then tell the clients what was in the message, making the SMS intervention somewhat complicated.

### Interviews with clients and family members

At the follow-up after the intervention, 11 clients and 11 family members (i.e., seven daughters, one son, one father, one mother and one niece) narrated their experiences of the SMS service. Almost all participants were positive to the SMS-support and the quotes below are illustrating their descriptions/experiences in their narratives. One client expressed it like this: *“I liked it – it kept me busy, I finished the work I was supposed to do”*. She continued describing what receiving the SMS meant to her in her everyday life: *“if this SMS had not, if they had not come … it would get me like that, I would not know that I need to practice these things on my own”.*

Another client agreed and expressed it like this*: “I would like to have more SMS, yes* [Clapping hands] *so much so much so much so much so much. I tell you those, it kept me very busy”.*

Other clients mentioned positive effects like *“Quicker service and contacts”, “The SMS-service has helped me in my everyday life”, “We are working towards the goals, we were reminded”.*

There were also clients mentioning that they would lack the SMS-reminders after the study period, like “*what will I do now when the SMSes will not come any more*”. According to the OTs, similar comments were given by a majority of the clients.

The family members narrated about many different target activities in the intervention and how they were assisting their family members (the person with stroke) in performing them. They also described the SMS-reminders regarding which activities they had decided to focus on during the rehabilitation period. Most of the family members were enthusiastic about the rehabilitation and the use of the mobile phone as a tool in rehabilitation. One family, for example, was so inspired by the opportunity to part in this intervention that they bought new phones so all of them could take part. The same family expressed that they liked being a part of the rehabilitation but that it sometimes was really demanding, that the physical training they did in between was “painful” and that “we practice at home because it is a must”. This family member described the SMS-reminders and follow-ups as difficult to handle and that they were too many. She said:“ *We reply, we do it because it is a must”.*

At the same time the same family member showed appreciation over the SMS-reminders. The SMS kept the family active. She described it like this: *“Sometimes we were bored, but now she (the mother) is not bored. She makes her life to be busy, good!”*

Another family member expressed that the best with the reminder system was that it included follow up to see if it worked and that the family member benefitted from it. She therefore appreciated the SMS-follow ups and phrased it like this: *…*“ *because in that way you show that you are concerned about the recovery of the client and follow-up. If you don’t follow-up you do not care. When you don’t care they are not interested”.*

Moreover, a daughter of another client also mentioned that she felt that the F@ce™ program and the SMS-reminders supported her in her work with, and worries about, her mother. The fact that the system automatically should tell the OT to call her mother if anything did not work, strengthened the daughter.

### Interviews with the participating occupational therapists

The four OTs participating in this project were all positive to the SMS-based reminder system. A number of advantages of the SMS-reminder system were identified from the answers like (based on quotes from the questionnaires):

- An SMS service like the one tested was seen as a good way to remind clients to perform daily activities to reach rehabilitation targets
- The OTs reported that the clients stated that the SMS system helped them to believe that someone really cared about them. Especially the reminder function was seen as very positive sign of that “someone” really cared about their health
- OTs also found the SMS system to be a good way to reach out to many clients regardless of the distance
- OTs also found the system to compel both the clients and the family members to adhere to the rehabilitation process
- It was also indicated by the OTs that the clients liked to monitor their own performance as they needed to send SMS rating their performances of the set targets
- The SMS system encouraged both clients and their family members to work together for the common rehabilitation goal

However, also a number of challenges were identified by the OTs:

- It was expensive to send and receive international SMSes
- Some clients struggled to send acceptable SMSes, since the system only accepted answers in the form of Figs. 0–5, some clients tried to answer in text, which did not work. When this did not work, they could become discouraged
- Some elderly clients were not familiar with SMS at all, and were only used to use their phones to call someone up
- Using the older type of mobile phones with buttons seemed to be challenging for some users, since they did not get an overview of what was sent and received (which can be more easily visualized on a Smart Phone)

### OT questionnaire

All four OTs filled in the questionnaire. The OTs were in general very positive to the SMS reminder system. Please refer to Table 1 for details.

**Table 1:** Answers from the OTs on the questionnaire

Question	Very much/Completely agree	Much/Agree	A little/To some extent	Not at all	Comments
Do you think the SMS-service has helped your clients in their rehabilitation?	2	2			
Do you think the SMS-service has helped your clients in their everyday life?	1	3			
Did you run into any technical problems with the SMS service?	1		3		Clients could not always receive messages
Using the SMS-service was an asset in the rehabilitation of the clients?	2	2			
Do you think the SMS-service has helped you in the rehabilitation with your clients?	3	1			
Would you recommend this SMS-service for others to use in the rehabilitation of clients?	4				
What was the best thing with the SMS system?	It enables clients to work out their objectives. They get to connect to therapists too. On spot. Gives a quick feedback. The time was good for reminding. It helped the clients to remember. To get the clients to work against the set goals.				
What was the most troublesome thing with the SMS-reminder system?	When they do not get feedback from the server (messages not going through) Sometimes, inconsistence: the therapists did not know exactly how the SMS came and looked like in order to advice the client better in how to use them The reply format on the afternoon questions was not easily understood				

## Discussion

In this pilot study, a SMS-based reminder system for supporting the rehabilitation process of post-stroke clients in Uganda was developed, implemented and evaluated regarding technical usability and user opinions. A special focus was to investigate if a relatively simple reminder system, based on pure text SMS-messages, could be developed and used, to overcome the challenge of that most clients in Uganda lack both access to modern Smartphones as well as to the Internet.

### SMS reminder system issues

It was found that a SMS-based reminder system could be developed and implemented rather straightforward and easy. However, a number of technical issues with this pilot system were also revealed. One was that SMS-messages only can handle 150 characters, and since there were three daily activities for each client, the reminder texts needed to be short. However, since none of the clients complained about the short messages, we believe that this mode of communication was acceptable.

In the future, where more persons probably will have access to smart phones also in low income countries, this issue will be less prominent.

The issue with that no local telecom operator supporting computer-based SMS-messaging API services could be identified in Uganda, led to the drawback that all messages needed to go from Sweden to the clients in Uganda, and the same with their responses on the evening surveys that were needed to be sent back to Sweden from the clients’ local phones in Uganda. This might also been the reason for that one of the clients did not receive the SMS-messages over a period of time. Even if the financial drawback of sending and receiving SMSes to and from Sweden was solved via pre-paying “air-time” for the participants, it is obviously not a perfect solution. This might also been the reason for that some clients had some other related issues (but which were solved). In a future reminder application, a local SMS API-service need to be set-up and connected to a local phone number and a local server. As far as we understand it, today this is possible also within Uganda.

A third issue was that some clients were unable to read and answer the SMSes sent out. This could be due to their post-stroke problems or to other reasons. In many cases, this challenge was solved via support from the clients’ family members, but this solution is not optimal. In the future, this problem might be eased by using tablets instead, where the text can be displayed in larger size and the “answer buttons” (soft touch-screen buttons on a tablet) can be made bigger. However, this will probably be an issue to look into in future studies. Post-stroke clients in any country often have both limited motor and cognitive abilities, and new smart solutions need to be developed in the future to overcome such problems.

Regardless of these challenges, we see it evident that a mobile phone-based reminder system can be implemented with rather small resources even in low income countries like Uganda. To the best of our knowledge there are no similar SMS-based reminder systems in Europe, which makes us believe that similar systems could be of use also in high income countries.

### The central SMS server system

As was shown in Figs. 1 and 2, the central SMS server system was used to both enter participant data, their related daily activities, record the client answers, automatically tell the local OT if a client was not answering, as well as to store and display follow-up data. This feature, even if not fully used in this pilot study, can probably be used in future similar projects to help clinicians to follow-up clients over longer terms.

Additionally, such a central system can also serve researchers with aggregated data for both individuals and groups of clients.

### Clients and family members

When asking the clients and their family members about their opinions of the SMS-reminder system, all were quite positive and indicated that this system made the rehabilitation quicker and better. The finding that some clients did not read and send back their responses on the SMS-messages themselves, but relied on that a family member assisted them, is somewhat challenging. It could be interpreted as a sign of that the SMS-reminders were difficult to handle. But on the other hand it can also be a sign of the culture to involve the family and that the family wanted to actively participate in the rehabilitation of the family member who had had a stroke. Moreover, we interpret the positive attitudes to the SMS-reminders as an indication that the mode of communication through SMS was well received, even if this was a completely new way of communication between clients and clinicians for all participants.

However, since this was a very limited study during a limited period of time, we have no hard evidence on that the rehabilitation results were really improved by the SMS-intervention. In the overall study [28], there was a significant difference detected between the IG and CG in changes between baseline and follow-up for the primary outcomes COPM performance component and self-efficacy, in favour of the F@ce™ intervention [27, 28]. Moreover, since rehabilitation measures after stroke often are based on that the clients themselves should feel that they make progress, we believe that the SMS-reminder system supported the clients in this way anyhow. Additionally, since more than one client expressed worries what should happen now, when they did not receive the reminders, we think that there was a real positive effect by the reminders. However, since the results are only based on clients’, OTs’ and family members’ expressed beliefs, we cannot guarantee that the SMS-reminders were resulting in objectively improved physical and cognitive health of the clients. Future studies should look into the possibility to more in detail assess the use of this type of reminder systems and their possible impact on the health of the clients.

### Occupational therapists

When interviewing the OTs participating in this study, their opinions were very positive as well. All four of them believed the SMS-service to be supportive to their clients in the rehabilitation process. All of them also indicated that the SMS reminder system supported their clients in their everyday life. This is to our mind good signs of a potential use of similar systems in the future, as well as in line with findings by Perri-Moore et al. [5] who mentioned that “Automated technology may reliably assist clients to adhere to their health regimen, increase attendance rates, supplement discharge instructions, decrease readmission rates, and potentially reduce clinic costs”.

The OTs also noted similar challenges as described above, regarding a number of technical issues. These included sending/receiving international calls, connectivity issues and problems reading the small screen etc. The fact that some clients were reported to have troubles with answering the evening SMS in Figs. 0–5 (and not in text) can be seen as an indication of that the instructions to the clients should have been more clear. But again, as have noted, a SMS-based system only allows short messages of 150 characters, so if the instructions should be longer, another type of communication mode might be needed like SmartPhone or Tablet apps or similar that allows longer texts. But such solutions also require access to Internet, which is not a standard for many people in low income regions. Moreover, the OTs interviewed also indicated that they would recommend this type of SMS-based system to others to use in the rehabilitation of clients, and that they believed that the reminder system helped their clients to improve.

### Overall results

Thus, taken together the findings support each other and are not just the opinions, but rather the common view of most participants involved in this study. Both the therapists and the clients and their family members were supportive of this type of mobile phone supported rehabilitation processes. However, as mentioned above, we cannot guarantee that the SMS-reminders were resulting in objectively improved physical and cognitive health of the clients. Other limitations of the study is that we only targeted Uganda and post-stroke clients linked to one hospital only. However, as many low-income countries often face similar issues with regard to limited access to technology, restricted financial capabilities as well as limited access to health care professionals, we believe that this study is a good example of what can be done in such areas and thus, we believe that the results should be applicable for also other low income regions of the world.

## Conclusions

This study, even if it was a limited pilot study, resulted in a number of conclusions, including that it was possible to develop and implement a SMS-based system for reminding clients post-stroke about the daily target activities, as well as to survey the clients’ for their estimation of the result of the activities. The system could also be used to automatically warn OTs about clients that were not able to perform the activities or who did not answer the survey questions. All clients seemed to appreciate the SMS-reminders as well as the evening survey about their success rate. Family members also seemed to see the SMS-reminder system as valuable. Involved OTs indicated the SMS-reminder system as a good tool to support rehabilitation after stroke saw great clinical possibilities of using the reminder system. The text-only based reminders was a good way to solve issues of that nearly all clients lacked smart phones and internet connections. A number of technical issues were identified, most of them due to the need to use international calls for the SMS-messages. Finally, if a similar system should be implemented in the future, all messages should be based on local communication set-ups.

## Additional file

Additional file 1: The Questionnaire to the OTs. (DOCX 14 kb)

## Data Availability

If our manuscript will be accepted for publication we are unfortunately unable to make the client data available publicly due to our responsibility to protect the confidentiality of the participants. This would also violate the ethical permit. As our OT participants were selected from a small total population of occupational therapists in Uganda, in making this information public we would be unable to ensure their anonymity, and would violate the conditions under which they agreed to participate in the study.
