# Supplementary material for: User evaluation of a novel SMS-based reminder system for supporting post-stroke rehabilitation
Source: BMC Med Inform Decis Mak. 2019 Jul 3;19:122. doi: 10.1186/s12911-019-0847-3 (PMC6610841; doi:10.1186/s12911-019-0847-3)
Supplement: Supplementary file 1 — The Questionnaire to the OTs. (DOCX 14 kb) [file 12911_2019_847_MOESM1_ESM.docx]

# Appendices

Appendix 1, the Questionnaire to the OTs

1. Do you think the SMS-service has helped your clients in their rehabilitation?

□ Very much □ Much □ A little □ Not at all

Comments:

1. Do you think the SMS-service has helped your clients in their everyday life?

□ Very much □ Much □ A little □ Not at all

Comments:

1. Did you run into any technical problems with the SMS-service?

□ Very much □ Much □ A little □ Not at all

Comments:

1. Using the SMS-service was an asset in the rehabilitation of the clients?

□ I completely agree □ Agree □ To some extent □ Not at all

Comments:

1. Do you think the SMS-service has helped you in the rehabilitation with your clients?

□ Very much □ Much □ A little □ Not at all

Comments:

1. Would you recommend this SMS-service for others to use in the rehabilitation of clients?

□ Very much □ Much □ A little □ Not at all

Comments:

1. What was the best thing with the SMS-reminder system?

Comments:

1. What was the most troublesome thing with the SMS-reminder system?

Comments:
